# The Prevailing Sentiment on the Consultation Question

**Published:** 1882-11

**Authors:** 


### Editorial.

### The Prevailing Sentiment on the Consultation Question.

The Secretary of the Medical Society of the State of New York has added, as a postscript to a circular in which attention is directed to the transactions of 1882, the following:

"Every person receiving the circular, is requested to send by postal card to the Secretary, a brief message, over his signature, stating whether he does or does not *personally* approve of the plan of leaving to the judgment of the individual physician, whether he shall or shall not meet, in consultation, legally qualified practitioners, not of his own school in medicine. A general compliance with this request will show what is the prevailing sentiment of the medical profession of the State, on the consultation question. All communications will be treated confidentially, if so desired."

It occurs to us to inquire the authority from which this request comes, and the motive inspiring it. Has the State Medical Society become convinced from the experience of its delegates at the American Medical Association, at St. Paul, that the sentiment of the profession throughout the country is opposed to the New Code? Is it the object of its officers, in adopting this method, to ascertain "the prevailing sentiment of the medical profession of the State" to strengthen the State Society in the position it has assumed on ethical questions, and to fortify it against such attacks and rebukes as the profession elsewhere are inclined to make? This circular anticipates the annual meeting of the State Society to be held in a few months, to which delegates are to be elected by the County Societies, who are presumed to express the sentiments of the constituencies that they represent.

We have devoted considerable space, in a previous number of the Journal, to an able discussion of the New Code. The arguments, pro and con, were courteously presented. We are inclined to think that a longer period of incubation is necessary than the State Secretary allows before the "prevailing sentiment" can be formulated upon so important a subject. If the New Code is based upon sound principle, the time intervening before the annual meeting, will bring forth a more settled purpose of adherence to its previous action than can be formed at this early day.

In the circular from which we take the postscript above referred to, is the announcement that the transactions for 1882 may be obtained of the Secretary. The volume of 500 pages contains many very able articles, and a verbatim report of the discussions. We are compelled to offer a criticism which we feel is deserved, although not ventured with any captious spirit. A work containing the medical thought and deliberations of so able a body as is represented by our State Medical Society should be presented in a better dress. The paper and the type are inferior, and the appearance of the volume is far below the standard now maintained by our leading publishers of medical works. We hope future editions will be improved and the volume made to correspond, in our libraries, with other standard works in its typographical execution.

